# Long hypocotyls facilitate deeper sowing to improve canola establishment in a warming climate

**DOI:** 10.1007/s00122-026-05396-2

**Published:** 2026-11-04

**Authors:** Matthew N. Nelson, Mark Cmiel, Alexander Zwart, Trijntje Hughes, Karen Treble, Andrew Toovey, Ian Greaves, Jose M. Barrero, Andrew L. Fletcher, John A. Kirkegaard, Greg J. Rebetzke

**Affiliations:** 1 CSIRO Agriculture and Food, Floreat, Perth, Australia; 2 CSIRO Agriculture and Food, Black Mountain, Canberra, Australia

## Abstract

***Key message*:**

**Deeper sowing of canola into cooler, moist soil could improve crop establishment in stressful conditions. We demonstrate cultivars with long hypocotyls emerge better from deeper sowing than short hypocotyl cultivars.**

**Abstract:**

Poor seedling establishment is a major constraint to canola (*Brassica napus* L.) productivity globally. Optimum seeding depths are commonly 1–2 cm under favourable soil moisture and temperature conditions., However, drying autumn conditions with climate change coupled with increasing farm size often results in canola sown earlier when the soil surface is hot and dry. Deeper sowing (> 3–4 cm) into stored water and more stable temperature allows for timely establishment of early sown crops. Under favourable moisture and temperature conditions, current cultivars establish well at shallow sowing depths but emerge poorly when sown deeper. This study investigated the potential to breed canola cultivars with longer hypocotyls to improve emergence from deep sowing as a strategy to adapt to earlier sowing under climate change – a concept demonstrated successfully with longer coleoptile wheat. A diverse panel of 255 open-pollinated accessions was phenotyped using high-throughput laboratory assays for hypocotyl length and cross-sectional area. Genome-wide association analysis revealed hypocotyl length was under polygenic control, and several significant marker–trait associations were identified. Emergence was validated in multiple field environments with both conventional (2 cm) and deeper (5 cm) sowing. Increased hypocotyl length was strongly associated with improved emergence from deep sowing, supporting its value as a breeding target to improve canola establishment under increasingly variable and challenging growing conditions.

**Supplementary Information:**

The online version contains supplementary material available at https://doi.org/10.1007/s00122-026-05396-2.

## Introduction

Canola (*Brassica napus* L.) (food/feed-grade oilseed rape) is a globally important oilseed crop, cultivated across temperate regions for its edible oil and protein-rich meal. In addition to its economic value as an oilseed crop, canola has an important agronomic role in temperate crop rotations by suppressing grass weeds and breaking disease cycles in intensive cereal systems (Kirkegaard et al. 2020). However, field establishment remains a major challenge in many canola production systems due to a range of biotic and abiotic factors (Nelson et al. 2022). Establishment rates often fall below 60% of viable seeds, resulting in reduced yield potential, increased weed pressure, and, in severe cases, the need for costly resowing (French et al. 2016; Harker et al. 2012; McMaster and Baxter 2019; Nelson et al. 2022).

The problem of poor canola establishment is exacerbated by climate change, increasing farm sizes and evolving farming practices. Furthermore, the economic risk associated with poor canola establishment is amplified by the high cost of seed relative to other temperate crops. In rainfed, water-limited environments, canola cultivars are bred with a targeted optimal flowering window specific to different cropping regions (Lilley et al. 2019). Establishing crops earlier to achieve optimum flowering times provides physiological advantages to improve water-use efficiency, by rapid ground coverage to reduce direct evaporative water loss, increased growth and transpiration efficiency during cooler autumn conditions and avoidance of heat and drought stress during the critical period of yield determination (Kirkegaard et al. 2020, 2016). Consequently, earlier sowing into warmer, drier soils is increasing, particularly in regions with Mediterranean or continental climates (Fletcher et al. 2016). Under these conditions, shallow sowing (c. 1 – 2 cm) exposes seeds to surface temperature extremes and increases the risk of rapid surface-soil drying and “false” seasonal breaks when follow-up rainfall fails and hot, dry soils risk seedling death. In addition, the increase in farm size in many major canola cropping regions due to economic efficiencies of scale means that sowing occurs earlier to complete the entire crop sowing program in a timely way. Under these earlier sowing scenarios, deeper sowing (c. 4- 5 cm) offers agronomic advantages by improving access to sub-surface soil moisture stored from summer rain, and buffers seedling emergence against high surface temperatures and rapidly drying soil to achieve timely and reliable crop establishment. However, the emergence and establishment of current commercial canola cultivars is known to decline significantly with increasing sowing depth (Brill et al. 2016; Malhi and Gill 2004).

Seedling emergence from deep sowing is influenced by several factors including seed size and vigour, and the elongation capacity of the hypocotyl—the organ connecting the root and emerging cotyledons (Nelson et al. 2022). In *B. napus*, the hypocotyl is the primary organ responsible for seedling elongation through the soil to the surface. Its length and strength are critical traits for successful emergence, particularly under deep sowing or marginal moisture conditions (Kirkegaard et al. 2020). The recent development of long coleoptile wheat cultivars provide an insightful example of how breeding for longer underground organs can dramatically improve emergence rates from depth compared to conventional cultivars with substantial grain yield improvements (Zhao et al. 2022). Canola has a comparatively small seed and therefore sowing depth is limited. It is expected then that even a modest increase in suitable sowing depth would have a greater marginal advantage in canola compared to a large-seeded cereal.

This study describes research efforts exploring the potential to develop long-hypocotyl canola to improve establishment with deep sowing. The strategy included the development and application of a new high-throughput phenotyping protocol for canola hypocotyl traits, genetic analyses of a large and diverse canola diversity panel, and field validation experiments characterising seedling growth traits and evaluating improved canola establishment resulting from increased hypocotyl length. The work forms part of a broader effort to provide canola breeders with traits to adapt canola to increasingly variable and challenging growing conditions. The research tested two hypotheses: (1) there was genetic variation in canola hypocotyl length and molecular markers linked to this trait, and (2) the long hypocotyl trait would improve canola establishment with deep sowing.

## Materials and methods

### OzCanola diversity panel seedlot characterisation

These experiments utilised germplasm from the *B. napus* OzCanola diversity panel, which comprised 778 *B. napus* open-pollinated accessions capturing species-wide diversity from 37 countries and all crop and maturity types (Zheng et al. 2026). A subset of 255 accessions was selected that included the major crop types, genetic diversity groupings and broad geographic origins (21 countries), while ensuring a strong representation of historical germplasm relevant to Australian conditions (40% of accessions) (Table 1; Table S1). Spring oilseed types were the primary focus (58%), then winter (27%) and semi-winter (9%) oilseed types. Included were 11 non-oilseed types and one synthetic variety.

**Table 1:** Regions of origin of 255 *Brassica napus* accessions (a subset of the ‘OzCanola’ panel) used in this study

Region of origin	Number of accessions	Representation
Oceania	101	40%
Europe	87	34%
Asia	40	16%
North America	23	9%
Unknown	4	2%
**TOTAL**	**255**	**100%**

To minimise maternal environment confounding genetic differences on seedling traits, seedlots were generated under favourable conditions for growth and development in a common garden seed nursery at the CSIRO Ginninderra Experimental Station, Australian Capital Territory, Australia (35° 11′ 45″ S, 149° 05′ 16″ E) during April to December 2019 (Zheng et al. 2026). Plants were grown in large pots with overhead irrigation supplied regularly to minimise moisture stress. Leaf samples from individual plants were obtained for genotyping (see below), and each pot individually bagged with large nylon mesh bags (Midco, Saint Louis, USA) to prevent cross-pollination with other accessions. Plants were harvested within the nylon mesh bags, threshed and seeds sieved using a slotted screen into four size classes: < 1.8 mm, 1.8 – 2.0 mm, 2.0 – 2.36 mm and > 2.36 mm diameter. Seedlots with sufficient seed (min. 20 g) were sub-sampled for seed quality testing using near-infrared spectroscopy (% oil and protein content, and glucosinolate content) and gas chromatography (fatty acid composition) by the New South Wales Department of Primary Industries and Regional Development’s (DPIRD) seed quality testing laboratory, Wagga Wagga, Australia.

### Developing a new hypocotyl phenotyping method

A preliminary experiment was undertaken to determine the duration (as thermal time (°Cd) using 0 °C as the base temperature) over which hypocotyls fully elongated for determination of maximum hypocotyl length. The goal was to determine the maximum extent to which hypocotyls could grow in the absence of light, and the hypocotyl length and thickness at that stage. As seed size may influence seedling growth traits, we restricted seeds for most accessions to a common size (2.00 – 2.36 mm sieve fraction) and weight (4.5 – 6.0 mg) and weighed each individual seed before sowing. Row-column spatial designs were created using Digger® (Butler et al. 2017) to assess potential row-column position effects on seedling growth.

Seeds were sown into soil at precisely 5 mm depth into Eazy Plug ® trays (11 × 7 array format, CT77; South Pacific Hydroponics, ACT, Australia) after first saturating with water and allowing to drain for two hours. Each Eazy Plug tray was placed between two black plastic trays and sealed with thick electrical tape to exclude all light and maintain a high relative humidity before placing into constant temperature cabinets. Six sets of trays each with 10 accessions (7 reps each) were grown at 16 °C and sampled at the following thermal timepoints: 160, 176, 193, 211 and 230°Cd post-sowing. One tray was sampled per timepoint except for the final timepoint where two trays were sampled.

Hypocotyl length was measured from the soil to the first node just below the cotyledon. Hypocotyl thickness was measured on the same seedlings using digital callipers (Whitworth, ACT, Australia) mid-way along the hypocotyl between the soil and the cotyledon and repeated immediately below the cotyledon. The seedlings were then oriented 90° and measured again. The mean radius was used to calculate hypocotyl cross-sectional area as CSA = πr^2^.

These experiments determined that hypocotyl elongation continued up to 210°Cd upon which hypocotyl length was maximal (Fig. S1). Appearance of first true leaves was closely associated with the end of hypocotyl elongation. The presence (1) or absence (0) of true leaves were assessed in the 193, 211 and 230°Cd harvests with mean scores of 0.25, 0.40 and 0.96, respectively (LSD = 0.09). The timing of hypocotyl elongation was the same among 10 accessions except for one slower growing accession (data not presented). These results indicated that the optimal thermal time duration for measuring hypocotyl-related traits was c. 230°Cd when hypocotyls were fully elongated and true leaves had appeared.

### Hypocotyl phenotyping of the OzCanola panel and current cultivars

The hypocotyl phenotyping method was applied to 255 open-pollinated accessions from the OzCanola diversity panel where seedlot production was closely controlled in a common seed nursery. Every accession was phenotyped at c. 230°Cd (15 days at 15 °C and 11 days at 22 °C) with at least two experimental runs per temperature and a minimum of three replicates per experimental run. The panel was phenotyped in two batches comprising 100 and 176 accessions with 21 overlapping accessions common to the two batches. Similarly, hypocotyl phenotyping was applied to a set of 41 current cultivars (30 hybrids and 11 open-pollinated; 39 spring and two winter types provided by Australian Grain Technologies, BASF Australia, NPZ Australia Pty Ltd, Nufarm Ltd, Nutrien Ag Solutions Ltd and RAGT Australia) using commercial seedlots provided by breeding companies using four replicates at the 15 °C temperature treatment only. These 41 commercial cultivars were used to test the relevance of the OzCanola panel to canola cultivars currently grown in Australia. Being commercially produced seedlots, we could not control maternal seedlot variation and hence these cultivars were analysed separately from the OzCanola accessions.

Phenotypic traits were analysed using linear mixed models fitted by restricted maximum likelihood (REML), using ASReml for R v4.2 (Butler et al. 2023). Fixed effects terms included accession/cultivar (genotype) and, where appropriate, temperature and their interaction to allow statistical significance testing of these main and interaction terms, with seed weight fitted as a run-specific covariate in each analysis. Random effects accounted for the experimental design structure (the ‘design model’) including run and tray nested within run, and heterogeneous residual variances modelled across runs (Oakey et al. 2006). The design models were then refined with further components to account for extraneous spatial effects (e.g. within-tray linear row trends or selected random row or range effects) when indicated by appropriate residual diagnostic plots and Wald and REML Likelihood Ratio tests. Best linear unbiased estimates (BLUEs) were obtained for genotype and genotype-by-temperature effects, and corresponding best linear unbiased prediction (BLUP) models were used to estimate variance components and generalised heritability (i.e. repeatability). In addition to comparisons between individual genotypes, countries of origin were also compared for mean differences in hypocotyl length and CSA using linear mixed models.

### Genome-Wide Association Study (GWAS)

The genetic control of hypocotyl traits was investigated using GWAS. Single nucleotide polymorphisms (SNPs) for 255 OzCanola accessions were obtained from Zheng et al. (2026). Briefly, 28,020 SNP loci were produced using the Illumina 90 K Infinium *Brassica* SNP chip (Katche et al. 2021; Mason et al. 2017) and by sequencing leaf transcriptomes (118,729 loci), and then assembled into a hapmap file comprising 146,749 SNPs in 255 accessions using the ZS11 reference genome (Song et al. 2020). The genetic structure of the population was estimated using two methods: principal component analysis (PCA) and fastSTRUCTURE (Raj et al. 2014). The GWAS analyses were conducted in GAPIT using GLM, MLMM, BLINK and FarmCPU models with and without adjusting for population structure (Lipka et al. 2012). Manhattan plots were generated using GAPIT with significance thresholds calculated using Bonferroni correction and false discovery rate (FDR) at P < 0.05. The phenotypic effects of significantly associated SNPs were calculated using regression analysis. Gene annotations were obtained from the BnIR database (Yang et al. 2023).

### Relating hypocotyl length to emergence from deep sowing in field experiments

Having identified extensive genetic variation for hypocotyl traits in the OzCanola panel, we determined the influence of hypocotyl traits on emergence from deep sowing (5 cm) in the field. We selected a subset of 21 diverse accessions from the OzCanola panel representing the entire range of hypocotyl lengths and other establishment-related traits. A common seed source and mean seed mass constrained to between 4.3 – 6.0 mg were used to reduce maternal environment variation on hypocotyl growth. Five commercial cultivars seedlots with a range of hypocotyl lengths were selected from the set of 41 commercial cultivars, with mean seed weights ranging from 3.0 – 6.3 mg (Table 2). Four commercial seedlots were used per field experiment (of which three were used in both 2021 and 2022) to benchmark OzCanola accessions against current Australian industry cultivars. Maternal seed effects could not be controlled for the commercial cultivars, which were produced independently of the OzCanola seedlots. Instead, average seed mass values were selected to match the mass values used for the OzCanola seedlots.

**Table 2:** Twenty-one diverse OzCanola accessions and five commercial canola cultivars (CC) used in field establishment experiments

Accession name	Country of origin	Crop / maturity type
6_06_3737	China	semi-winter
Abukumanatane	Japan	winter
ATR_Bonito	Australia	spring
Baltia	USSR	winter
Capitol	France	winter
CouveNabica	Portugal	swede
Haya	Japan	spring
Karoo-057DH	Australia	spring
Lifura	Germany	fodder
MadrigalxRecitalDH	GBR	winter
Norin	Japan	winter
Reston	Canada	spring
Samourai	France	winter
SlovenskaKrajova	Czechoslovakia	winter
Surpass400-024DH	Australia	spring
Tantal	France	spring
Tribune	Australia	spring
Willi	Denmark	spring
Zircon	Australia	spring
TowerBC5	Canada	spring
Quinta	Germany	winter
CC_02	Australia	spring
CC_12	Australia	spring
CC_16	Australia	spring
CC_20	Australia	spring
CC_21	Australia	spring

Country of origin and crop / maturity type information is provided. Further details for OzCanola accessions are provided in Table S1.

**Fig. 1:**
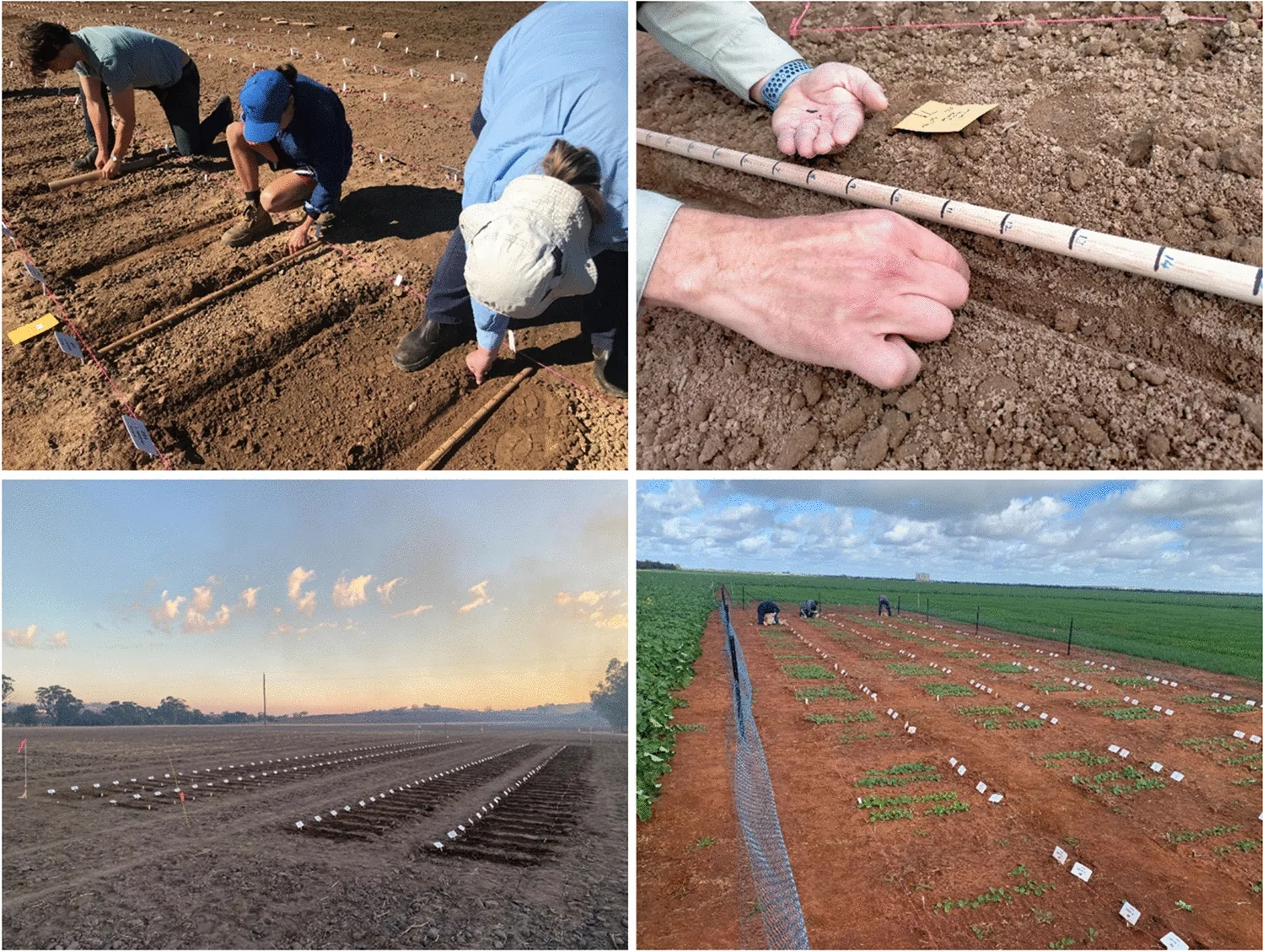
Field experiments to compare emergence of short and long hypocotyl canola genotypes from standard (2 cm) and deep (5 cm) sowing depths. Precisely measured rows (**A**), seed placement (**B**), in four replicated blocks (C and D). Photo credits: John Kirkegaard, Greg Rebetzke and Andrew Toovey

Field experiments were conducted at eight site × year combinations in 2021 and 2022 in Western Australia and New South Wales (Table 3). Sites were selected to represent a range of soil types and climates across the Australian cropping zone. Soil temperature loggers (Thermochron, Castle Hill, Australia) were placed at two diagonally opposite corners of each experiment at the two sowing depths optimal (2 cm) and deep (5 cm). Previous studies suggest emergence of current canola cultivars can be reduced by 50% at this greater soil depth (Brill et al. 2016). Experimental design at each site was a spatially optimised, split-block design comprising four replicates arranged in blocks with randomisation performed using Digger (Fig. S2). Accessions/cultivars (n = 25) were randomised with pairs of adjacent rows (whole plots) each containing 15 seeds sown at two sowing depths (2 and 5 cm) with 50 cm between the rows. Sowing depth was then randomised between the adjacent rows (split plots). Seeds in each plot were individually hand-sown precisely 6 cm apart before carefully covering with hand-firmed soil to provide an even surface and consistent soil depth above the seed (Fig. 1).

**Table 3:** Locations, soil types and dates of canola establishment field experiments

Site	Latitude	Longitude	Soil texture	Year(s)	Sowing date	Accumulated thermal time (°Cd)
Bejoording, WA	-31.38	116.57	Sand	2021	4-May	842
Boorowa, NSW	-34.45	148.69	Clay loam	2021	7-May	310
Griffith, NSW	-34.09	145.87	Sandy loam	2021	25-May	361
Kokeby, WA	-32.22	117	Sandy loam	2021	5-May	330
Boorowa, NSW	-34.45	148.69	Clay loam	2022	3-Jun	592
Fisher, WA	-31.91	116.71	Sandy loam	2022	11-May	607
Griffith, NSW	-34.09	145.87	Sandy loam	2022	25-May	557
Hillside, WA	-31.89	116.76	Sandy loam	2022	10-May	595

Accumulated thermal time at completion of each experiment was calculated using 0 °C as the base temperature. NSW = New South Wales; WA = Western Australia.

Seedling emergence was monitored 10 times at each field site between first emergence and completion of the experiment when most plants emerging from 2 cm sowing depth had reached the 4-leaf stage (BBCH 14). Photographs were taken of every full plot using measuring rods placed precisely along rows to indicate where seedlings were expected to emerge. Each photograph was examined and counts of emerged seedlings made for each date. Some seedlings emerged but did not survive to the end of the experiment due to pest or other damage; these were noted, and so emergence counts included all seedlings that had emerged, not only those that survived to the end of the experiments. At completion of the experiments (~ leaf 4 in 2 cm sowing), all rows were individually harvested and plant biomass (dry weight) recorded on a per-plant basis. The accumulated thermal time of each experiment was calculated using 0 °C as the base temperature.

The eight field experiments were first analysed individually and then as a multi-environment trial (MET) using linear mixed models fitted with ASReml for R v4.2 (Butler et al. 2023) in R v4.3.1. For the MET, due to the smaller number of lines grown in these trials, genotype, environment and genotype × environment interaction were fitted as fixed effects, with trial-specific block terms and spatially autocorrelated residual structures included where supported by prior single-trial analyses. Linear contrasts were estimated using predictPlus() in asremlPlus v4.4.13 (Brien 2023) and are reported as contrast BLUEs with 95% confidence intervals.

For the MET analysis, split-plot observations were aggregated to one value per whole plot, trial and response by calculating the 5 cm:2 cm ratio for emergence count and biomass per plant:

ratio = Y(5 cm)/Y(2 cm)

Biomass per plant was also analysed separately at each sowing depth by subsetting the data to 2 cm or 5 cm, thereby treating each depth-specific analysis as a randomised block design at the whole-plot level.

Residual diagnostics from single- and multi-environment analyses were used to assess heterogeneity of residual variance and the need for transformation. Square-root transformations were applied to biomass per plant ratio, biomass per plant at 2 cm and biomass per plant at 5 cm; contrasts for these responses were calculated on the transformed scale.

## Results

### OzCanola seedlot characterisation

Seedlots of 255 *B. napus* open-pollinated accessions were characterised for seed weight, seed quality and heterozygosity. Thousand seed weights ranged from 2.2—7.7 g. To minimise the effect of variation in seed size on expression of hypocotyl traits, experiments used the seed size fraction (2.00 – 2.36 mm) for most (n = 241) accessions. The remaining accessions had smaller seeds and so the 1.80 – 2.00 mm seed size fraction was used for these 14 accessions (Table S1).

The seed quality of 241 seedlots was measured using near-infrared reflectance and gas chromatography analyses; 14 seedlots had insufficient seeds for analysis (Table S1). The majority of tested seedlots (n = 129) were canola quality with erucic acid under 2% of total fatty acids and glucosinolates under 30 µmol / g of oil-free meal (10% moisture content). The remainder had single-high (n = 52) or double-high (n = 60) seed quality profiles.

The estimated heterozygosity of accessions (based on 146,749 SNP markers in one plant per accession) ranged from 0.1% to 31.6%, with a mean of 6.6% (Table S1) indicating that most seedlots were substantially inbred but that some accessions were genetically variable.

### Hypocotyl trait variation in OzCanola accessions

**Fig. 2:**
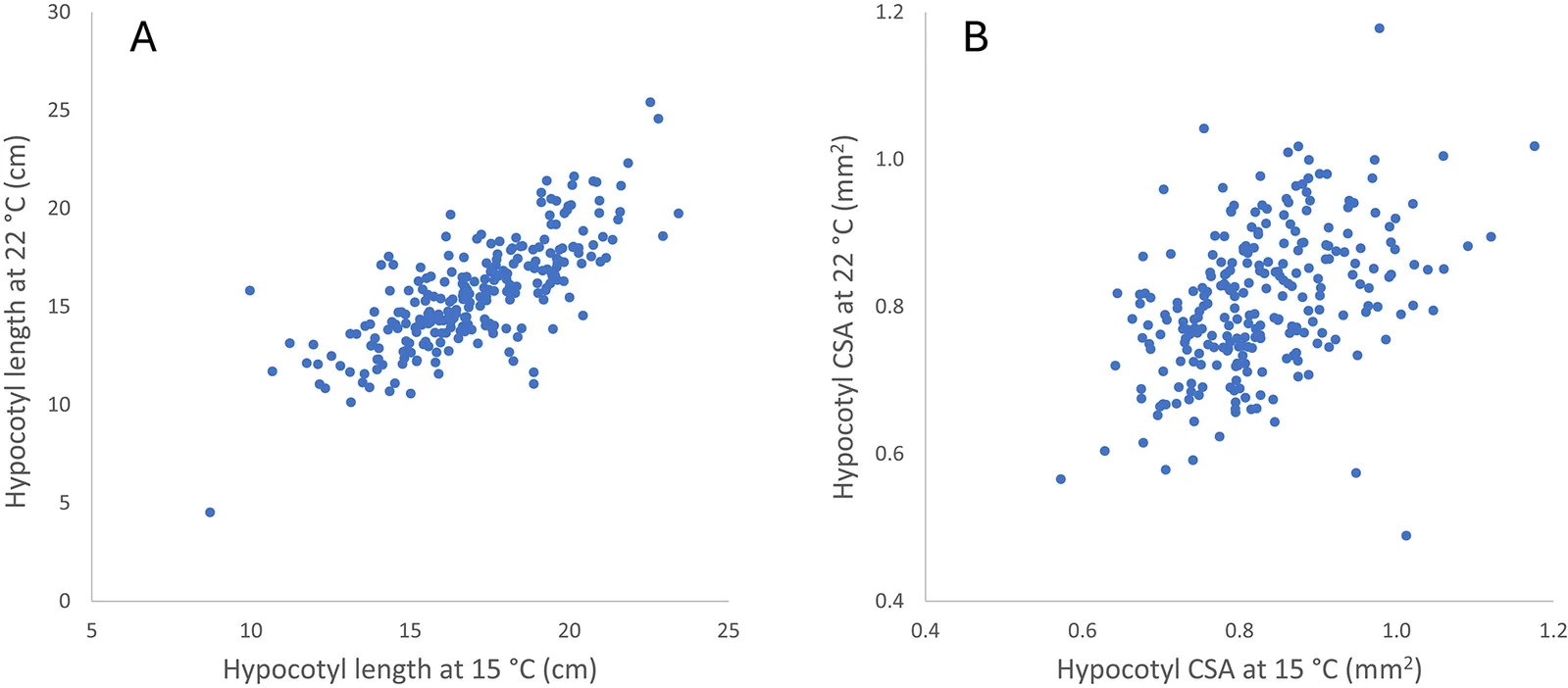
Hypocotyl length (**A**) and cross-sectional area, CSA (**B**) at 15 and 22 °C for 255 *Brassica napus* accessions measured 230°Cd after sowing

There was wide phenotypic variation for hypocotyl length and cross-sectional area (CSA) measured 230°Cd after sowing averaged across the 255 accessions of the OzCanola diversity panel (Fig. 2; Table S1). Mean hypocotyl lengths ranged from 8.7 – 23.4 cm (at 15 °C) and 4.5 – 25.4 cm (at 22 °C). Mean CSA ranged from 0.57 – 1.18 mm^2^ (at 15 °C) and 0.49 – 1.18 mm^2^ (at 22 °C). Wald’s tests confirmed that differences between accessions (genotype) was the largest factor (P < 0.001) for both hypocotyl traits, with high generalised heritability for hypocotyl length when seedlings were grown continuously at 15 °C (H^2^ = 0.846) or 22 °C (H^2^ = 0.845). Hypocotyl CSA was moderately heritable at 15 °C (H^2^ = 0.599) and 22 °C (H^2^ = 0.581). Hypocotyl length and CSA were negatively correlated both at 15 °C (r = − 0.457, P < 0.001) and 22 °C (r = − 0.418, P < 0.001).

The impact of temperature was highly statistically significant for both hypocotyl length (P < 0.01) and CSA (P < 0.001). The genotype × temperature interaction was also highly significant for hypocotyl length (P < 0.001) and CSA (P < 0.001). Nevertheless, hypocotyl length of accessions maintained similar rankings between temperature treatments (r = 0.756, P < 0.001; Fig. 2A), although CSA rankings were less well conserved between temperature treatments (r = 0.427, P < 0.001) (Fig. 2B).

**Fig. 3:**
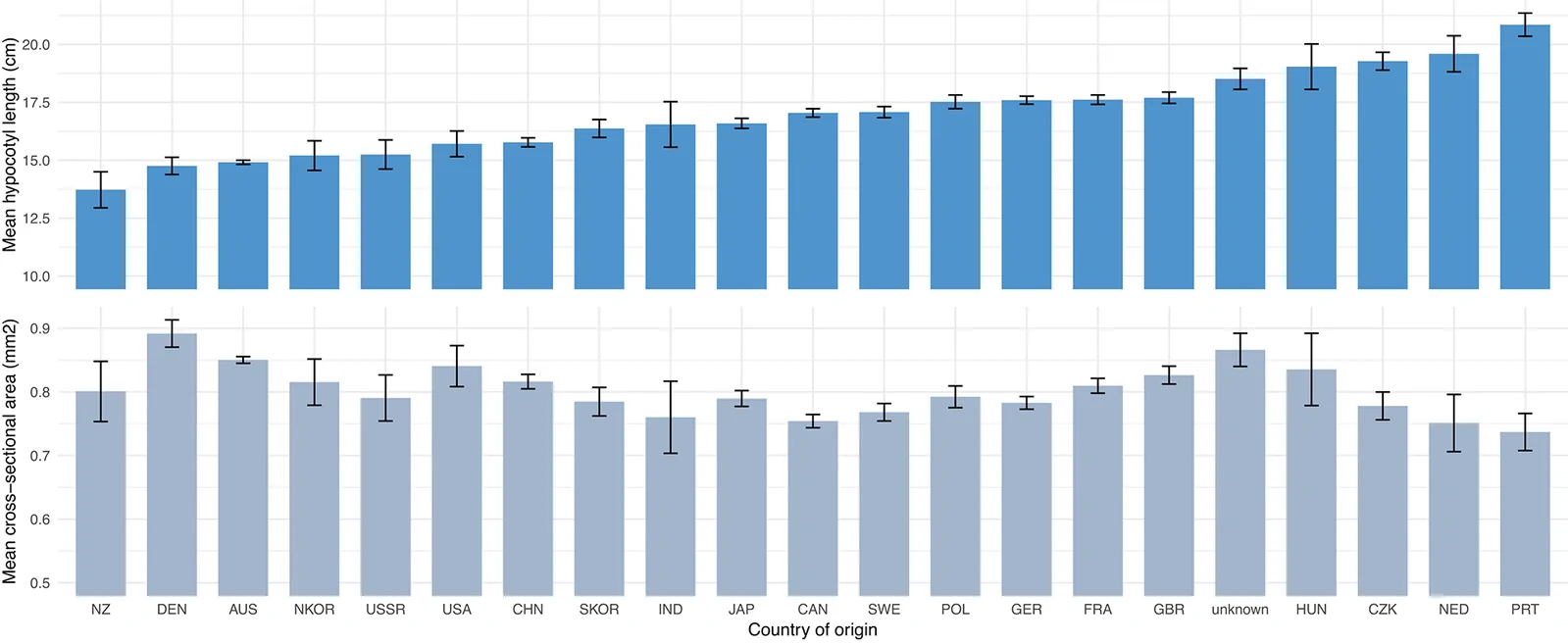
Mean hypocotyl lengths (upper panel) and mean cross-sectional areas (lower panel) for 255 OzCanola accessions from 21 countries. Results from 15 °C and 22 °C treatments were combined for this analysis. Error bars denote standard errors for country means. Average SED was 0.44 cm for hypocotyl length and 0.026 mm^2^ for cross-sectional area

Country of origin had a significant impact on mean hypocotyl length and CSA (Fig. 3). Countries represented largely with spring oilseed types tended to have shorter hypocotyls while countries represented by winter or vegetable types tended to have longer hypocotyls. By contrast, large among-country variation in CSA was not associated with any specific development or culinary type. The influence of vernalisation responsiveness and thermal requirement for flowering on hypocotyl length was investigated further using modelling parameters reported by Zheng et al. (2026) for a subset of 194 OzCanola accessions (Table S1). Long hypocotyls were associated with greater vernalisation requirement and longer thermal time to floral initiation (with and without saturating vernalisation), ranging from r = 0.277 to 0.408 (P < 0.001) for 15 °C and 22 °C treatments. Little or no association was observed between these flowering parameters and hypocotyl CSA.

Relationships between seed trait variation and hypocotyl traits across accessions were explored (Table S2). Increased seed oil content was positively correlated with increased hypocotyl length at both 15 °C (r = 0.276, P < 0.001) and 22 °C (r = 0.307, P < 0.001). Conversely, increased seed protein content was associated with reduced hypocotyl length at both 15 °C (r = − 0.237, P < 0.001) and 22 °C (r = − 0.260, P < 0.001). Neither oil or protein content were associated with variation in hypocotyl CSA. In contrast, canola quality traits (seed glucosinolate and erucic acid) were either uncorrelated or weakly associated with hypocotyl length and were weakly negatively correlated (correlations between − 0.129 and − 0.193) with hypocotyl CSA.

Finally, the representativeness of historic Australian accessions in the OzCanola panel was benchmarked against current commercial cultivars, which were mostly hybrids. When measured at 15 °C, the mean hypocotyl length of 41 current commercial cultivars was 15.1 cm (ranging from 13.0 – 19.1 cm; commercial data not presented). This compared to a mean of 15.7 cm (ranging from 10.0 – 19.7 cm) for 100 historic Australian accessions in OzCanola panel (Table S1).

### Exploring the genetic control of hypocotyl traits using GWAS

**Fig. 4:**
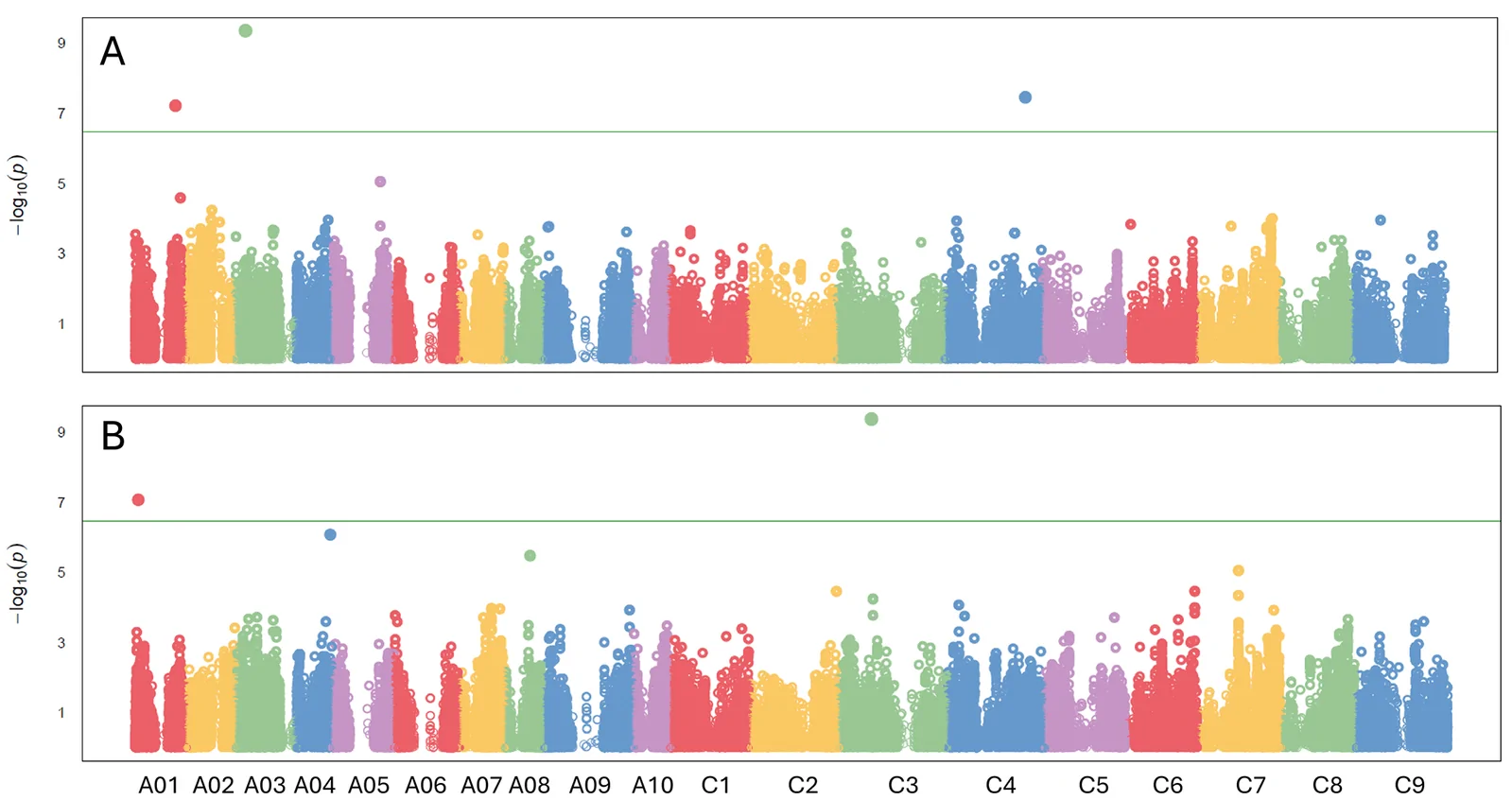
Manhattan plots for hypocotyl length measured at 15 °C (**A**) and 22 °C (**B**) treatments and 146,749 SNPs in 255 accessions across 19 *Brassica napus* chromosomes (A1-A10, C1-C9) detected using the BLINK model in GAPIT with adjustment for population structure using PCA5. Horizontal lines denote Bonferroni thresholds for statistical significance (P = 0.05)

Hypocotyl trait estimates (BLUEs) for 255 OzCanola accessions and a hapmap file comprising 146,749 SNP genotypes were mapped to the ZS11 reference genome. The genetic structure of the population was estimated using the first five principal components (PCA5) from a principal component analysis and K1-K7 groups defined by fastSTRUCTURE, both calculated using the same 146,749 SNPs. GWAS was performed using FarmCPU, BLINK, GLM and MLMM models in GAPIT with PCA5- and STRUCTURE-adjusted models as well as the base model disregarding population structure. The BLINK model with PCA5-adjusted data was the most effective combination to identify significant marker-trait associations for hypocotyl length: five SNPs exceed the Bonferroni threshold (Fig. 4), and six SNPs exceeded the less conservative FDR threshold (Table 4). Despite the high correlation between accession hypocotyl lengths at 15 °C and 22 °C temperature, there was no overlap of significant marker-trait associations shared by both treatments (Fig. 4A and B). No significant marker-trait associations were detected for hypocotyl CSA (results not presented).

**Table 4:** Significant associations of hypocotyl length measured at 230°Cd using 15 °C and 22 °C treatments and single nucleotide polymorphism (SNP) markers in 255 OzCanola accessions

Treat-ment	SNP locus	Chr. Name	Position, nt	SNP genotype^1^	MAF	P.value	P value (FDR-adjusted)	Effect, cm^2^	PEV, %	Gene, ZS11	Gene, Arabidopsis	Gene function
15 °C	Bn-A01-p21797129	A01	29,948,310	A/G	0.076	6.10E−08	2.97E−03	1.430	24.2	BnaA01G0322900ZS	AT3G18990	B3 domain-containing transcription factor VRN1
15 °C	Bn-A03-p6907743	A03	7,307,638	T/C	0.294	4.48E−10	6.55E−05	-1.182	19.4	BnaA03G0143600ZS	AT5G50720	HVA22-like protein
15 °C	BnaC04T0461000ZS_58794241	C4	58,794,241	C/T	0.420	3.52E−08	2.57E−03	-0.778	8.1	BnaC04G0461000ZS	AT1G64840	Protein of unknown function (DUF295; AtFDA3)
22 °C	BnaA01T0051100ZS_2741748	A01	2,741,748	A/C	0.100	8.47E−08	6.19E−03	-1.979	29.4	BnaA01G0051100ZS	AT4G32720	Phosphoprotein La protein; AtLa1
22 °C	BnaA04T0280400ZS_24575683	A04	24,575,683	A/C	0.400	8.30E−07	4.05E−02	0.611	5.9	BnaA04G0280400ZS	AT2G44120	Ribosomal protein L30/L7 family protein
22 °C	BnaC03T0353200ZS_23907983	C3	23,907,983	G/A	0.392	4.20E−10	6.15E−05	0.937	11.8	BnaC03G0353200ZS	AT3G04600	tRNA synthetases class I (W and Y)

Chr. = Chromosome; MAF = minor allele frequency; FDR = false discovery rate (P < 0.05); PEV = percent explained variance.

^1^ SNP genotypes are given as Reference (ZS11) / Alternative alleles.

^2^ SNP Effects are expressed as the effect of the Alternative alleles relative to the Reference (ZS11).

The six significant SNP-associated markers (FDR, P < 0.05) estimated genetic effects of between 0.61 and 1.98 cm for hypocotyl length, explaining 8.1—24.2% and 5.9—29.4% of variation in 15 °C and 22 °C treatments, respectively (Table 4). All six SNP markers were located in annotated genes with reported roles in Arabidopsis including ABA response (Bn-A03-p6907743), vernalisation (Bn-A01-p21797129), protein synthesis (BnaC03T0353200ZS_23907983), cell cycle regulation (BnaC04T0461000ZS_58794241) and ribosome function (BnaA01T0051100ZS_2741748, BnaA04T0280400ZS_24575683).

### Field validation of influence of hypocotyl traits on field emergence and establishment

#### Overview of field experiments

Each of the eight field experiments were concluded when 90% of accessions reached the 4-leaf stage (BBCH 14) at 2 cm sowing depth (Table 3). Seven of the experiments were considered successful; the exception being the Kokeby 2021 experiment, where waterlogging, weed competition and grazing by native animals together compromised the experiment. The Bejoording 2021 (sandy loam; Table 3) experiment experienced nutrient deficiency, which slowed plant development but otherwise produced robust data.

Fitting accession as a random effect (to allow estimation of genetic variances and heritabilities) revealed high heritability for seedling number emergence ranging from 0.793 to 0.852 across the seven environments. Biomass per plant (dry weight) from 2 and 5 cm sowing depths were adjusted logit-transformed to achieve normal distribution of data and BLUEs calculated. Heritability for this trait was similarly high across seven environments ranging from 0.760 to 0.899. We therefore had confidence to explore these results in detail.

#### Seedling emergence from conventional and deep sowing

Firstly, we confirmed that our approach of restricting seed size variation in seedlots was effective by finding no significant influence of thousand seed weight on seedling emergence success from optimal (2 cm) and deep (5 cm) sowing (Fig. S3). Restricting seed size removed a potentially confounding factor that may have masked true genotype hypocotyl and seedling emergence effects.

**Fig. 5:**
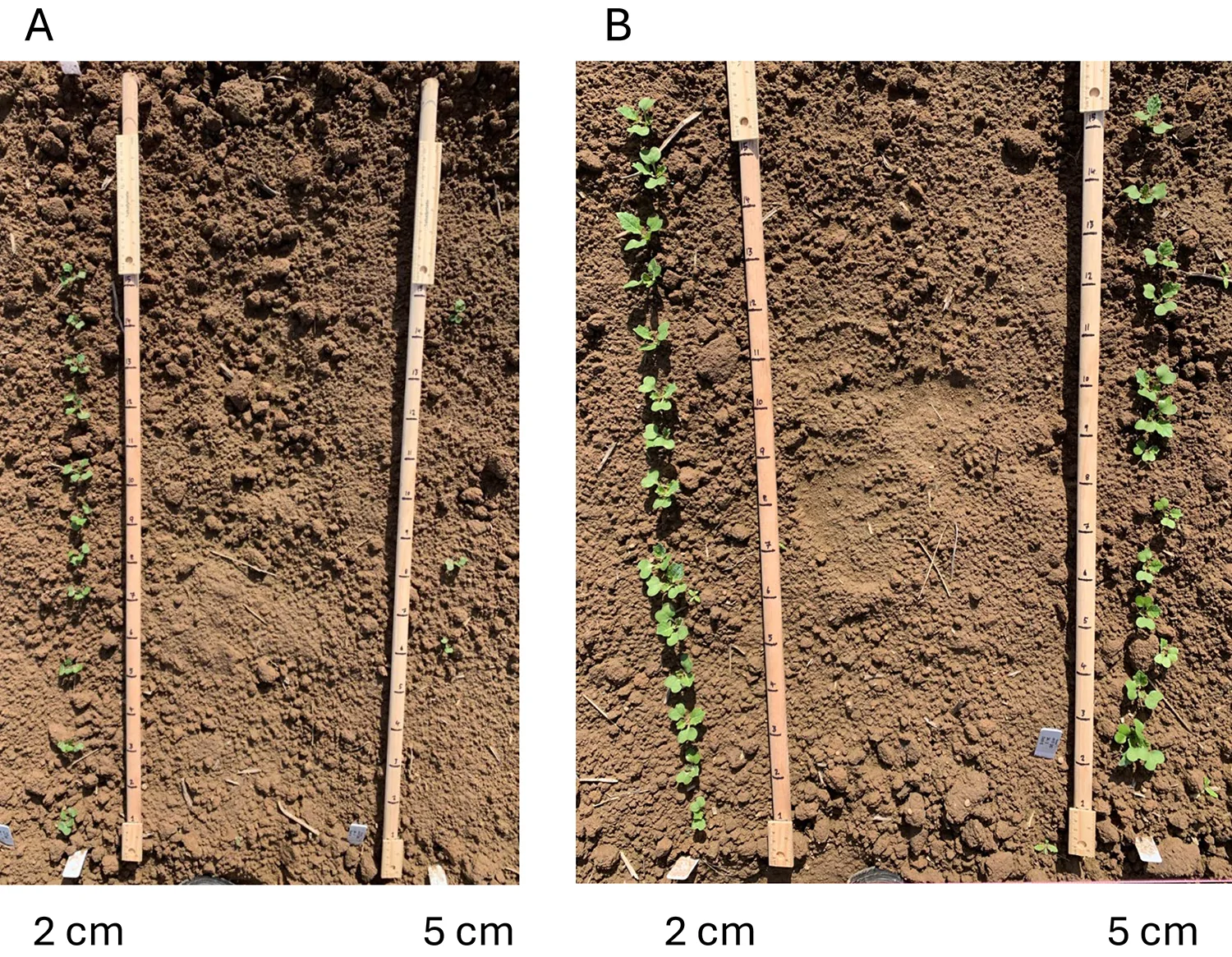
Contrasting responses of OzCanola accessions to deep (5 cm) compared to conventional (2 cm) sowing depths at the Boorowa field experiment in 2021. (**A**) a representative short hypocotyl, low vigour accession (Surpass400-024DH) and (**B**) long hypocotyl, high vigour accession (SlovenskaKrajova). Photo credit: John Kirkegaard

Maximum emergence counts (from 15 seeds sown) ranged widely across field environments and genotypes at both sowing depths. At the conventional 2 cm sowing depth, the maximum emerged count means for 25 genotypes ranged from as low as 3.2 – 10.5 (Bejoording, 2021) to 12.8 – 14.8 (Fisher, 2022) (Table S4). Emergence success was reduced at deeper sowing (5 cm) with genotypes differing significantly (P < 0.001) from 0.8 – 4.0 at Bejoording (2021) to 4.1 – 14.2 at Griffith (2022) (Table S4). Genotype differences in plant emergence at 5 cm were significant at six of the field experiments. Genotype x sowing depth was below the 0.05 significance threshold in one field experiment only (Boorowa, 2021). In other words, when analysing single sites, it was difficult to identify genotypes that responded differently to sowing depth treatment except for the Boorowa 2021 experiment where some genotypes clearly emerged better from deeper sowing than others (Fig. 5).

The interaction of genotype and sowing depth was explored further by calculating the emergence ratio (plant number at 5 cm / plant number at 2 cm) for each genotype at each field experiment. This trait ranged in generalised heritability from 0 (Hillside, 2022) to 0.736 (Boorowa, 2021) (Table S5). The six field experiments with non-zero heritability for emergence ratio were analysed together in a multi-environment trial (MET) analysis, fitting environment as fixed and genotype-environment interaction as random, to allow estimation of genetic variances and correlations between environments. Both positive and negative genetic correlations were observed between environments suggesting complex G x E interactions for this trait (Fig. S4). For example, emergence ratios for genotypes at the NSW experiments (Boorowa and Griffith in 2021 and 2022) were highly consistent. In contrast, results from the nutrient-deficient Bejoording (2021) experiment were negatively correlated with most of the NSW experiments.

**Fig. 6:**
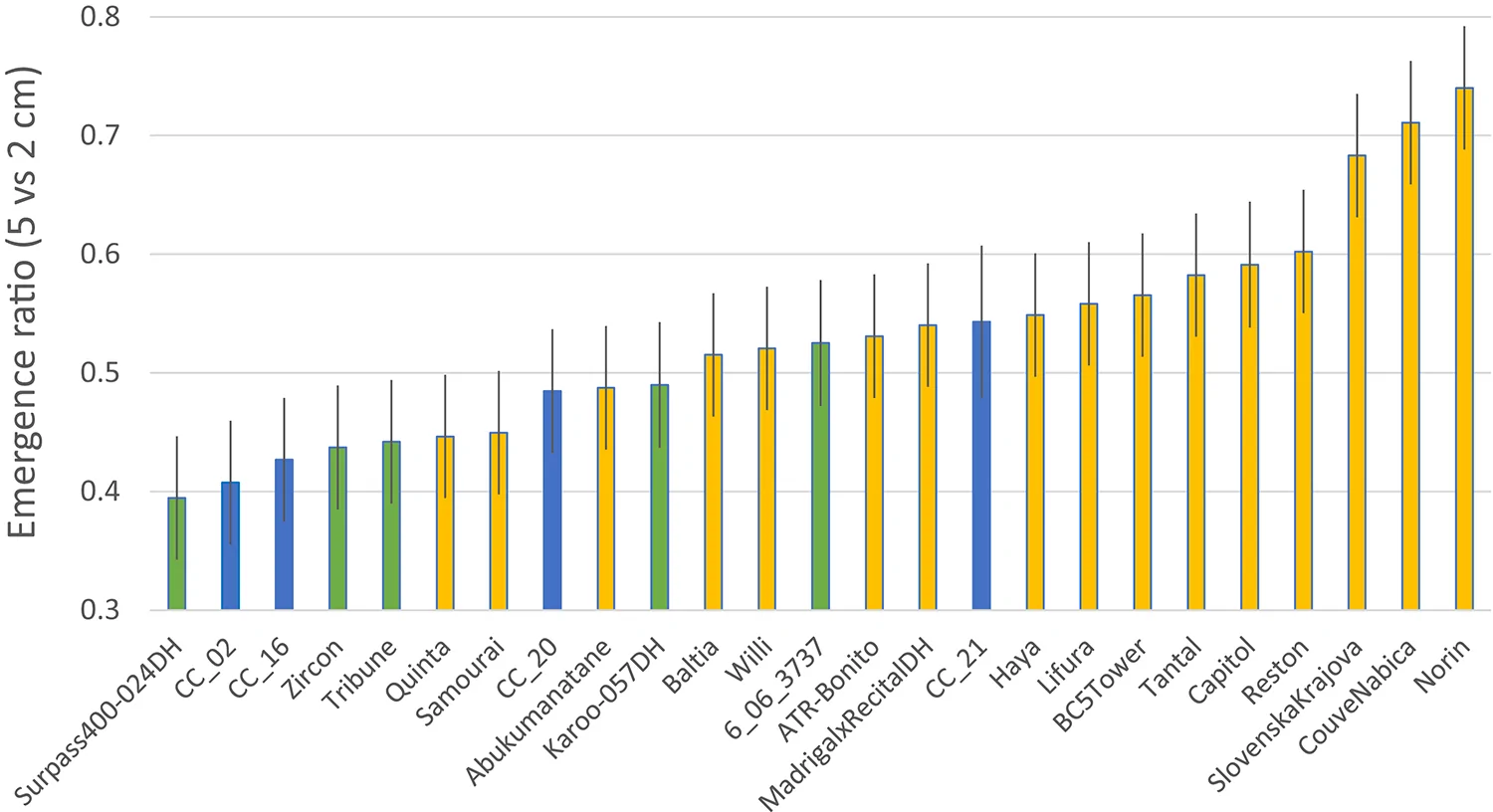
Emergence ratios (5 vs. 2 cm) for 21 OzCanola accessions (historic Australian cultivars in green, other international cultivars in orange) and four commercial Australian canola cultivars (blue bars) obtained by multi-environment trial analysis of six field experiments. Error bars denote one standard error

The combined emergence ratio BLUEs for each of the 25 genotypes (21 OzCanola international accessions and four Australian hybrid cultivars) across the six sites are presented in Fig. 6. It was notable that Australian commercial cultivars emerged poorly from deep sowing and significantly worse than the three best OzCanola accessions: Norin, CouveNabica and SlovenskaKrajova (Fig. 6).

**Fig. 7:**
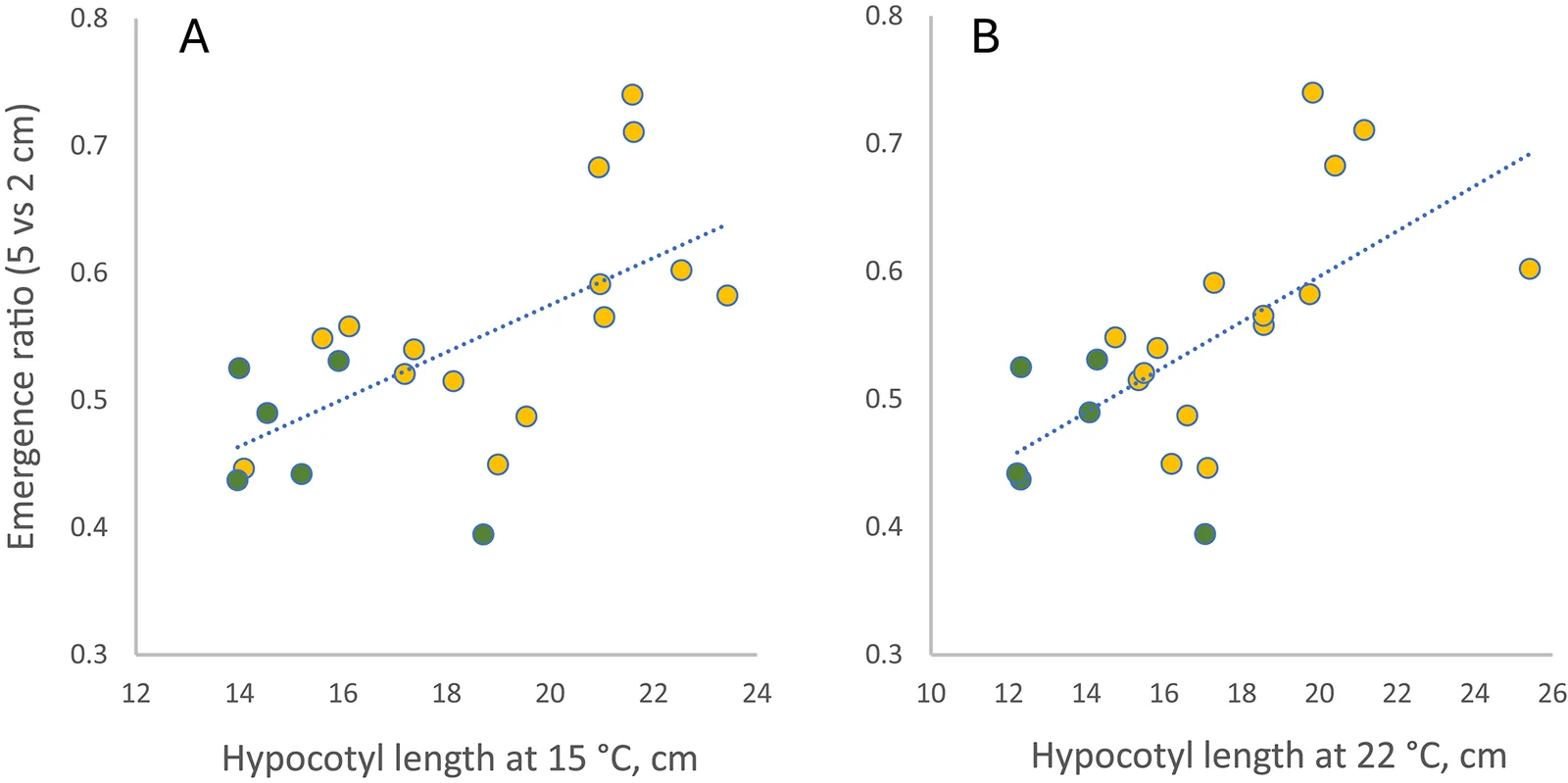
The relationship between emergence success of 21 OzCanola accessions from deep sowing (MET-BLUPs measured across six field experiments and expressed as emergence ratio, 5 vs. 2 cm sowing depths) and lab-based hypocotyl lengths measured at 230°Cd in (**A**) 15 °C and (**B**) 22 °C treatments. Six Australian historic cultivars are indicated by green filling with orange-filled points denoting international cultivars

Finally, the relationship between lab-based measurements of hypocotyl length and plant emergence from deep sowing in the field was assessed in 21 OzCanola accessions. The controlled environment-selected, long hypocotyl accessions emerged better from deep sowing than short hypocotyl accessions (Fig. 7), for hypocotyls measured at both 15 °C (r = 0.629, P = 0.002) or 22 °C treatments (r = 0.641, P = 0.002).

#### Biomass per plant

The relationship between thousand seed weight of sown seeds and mean biomass per plant (dry weight) at harvest was small in all field experiments except for a modest relationship at Bejoording in 2021 at both 2 cm (r = 0.520; P = 0.01) and 5 cm (r = 0.402, P = 0.047) sowing depths (Fig. S5).

Mean biomass per plant ranged widely across genotypes and field environments, and at both sowing depths. Sowing depth was the most significant factor in each experiment (P-values ranging from 5.67E−27 to 0.057) with deeper sowing reducing biomass in a largely uniform manner as indicated by significant correlations between sowing depths (Table S6; Fig. S6). The Bejoording 2021 experiment showed the smallest sowing depth effect (P = 0.057), presumably due to nutrient deficiency experienced by this experiment. There was a weak interaction of genotype x sowing depth at three sites (Boorowa, 2021; Fisher, 2022; Griffith, 2022).

There was no significant relationship observed between biomass per plant (for either 2 cm or 5 cm sowing depth) and hypocotyl length (measured at 15 °C or 22 °C); this was true for individual experiments and for the combined MET analysis (data not presented).

There was a positive relationship between biomass per plant at 5 cm sowing depth and maximum emergence at Boorowa 2021 (r = 0.574, P < 0.01), Griffith 2021 (r = 0.604, P < 0.01) and Griffith 2022 (r = 0.598, P < 0.01). No other relationship between these traits were observed at 2 cm sowing depth or in MET predicted values at either 2 or 5 cm sowing depths.

## Discussion

### Long hypocotyls as a strategy to improve canola establishment under warmer and drier sowing conditions

This study demonstrated that genetic variation for canola hypocotyl length can be phenotyped reliably with a new high-throughput, dark-grown controlled environment assay at ca. 230°Cd, and that this trait was strongly and positively associated with seedling emergence from deep sowing across multiple field environments (Fig. 7). The high-throughput assay was highly repeatable (generalised heritability of ca. 0.85) and across growth temperatures consistent with analogous methods developed in wheat (Rebetzke et al. (2007; 2012). The method is relatively low cost and does not require complicated equipment. The method has capacity to phenotype and potentially thousands of canola seedlings for hypocotyl length. Further, the strong genetic correlation for hypocotyl length at 15 and 22 $^\circ$ C is consistent with similar analyses in wheat (Rebetzke et al. 2016) where rank changes are small at cooler temperatures consistent with soil temperatures at sowing in many environments.

The greater emergence of long hypocotyl cultivars under deep seed placement aligns with known physiological principles in other crops. In cereals successful emergence with deep sowing is improved by a long coleoptile (Bathgate et al. 2025). In canola, it is the functionally analogous hypocotyl that is responsible for elongation and emergence of seedlings through the soil profile (Kirkegaard et al. 2020). Elongation capacity and maximum length is thus likely a critical determinant of establishment success under deep sowing. This would enable deeper sowing to overcome marginal moisture and temperature conditions when sowing early (Nelson et al. 2022). Earlier agronomic studies have shown that deep sowing offers improved access to sub-surface moisture but is rarely feasible with current cultivars because emergence success declines rapidly with depth beyond 3 cm (Brill et al. 2016; Harker et al. 2012; Malhi and Gill 2004). Consequently, our results provide a clear breeding target to overcome this agronomic constraint to growing canola.

The strong relationship between hypocotyl length and emergence success parallels recent breakthroughs in deep-sowing wheat, where long coleoptiles substantially improved emergence and early vigour under hot, dry sowing conditions (Zhao et al. 2022). Together, these findings indicate that selection for seedling elongation from the seed to the soil surface is a powerful strategy to increase crop resilience to increasingly variable climates (Flohr et al. 2021).

### Trait architecture and phenotypic trade-offs

Hypocotyl length and CSA measured in controlled environment assays exhibited high and moderate heritability, respectively. The negative correlation between hypocotyl length and CSA mirrors observations in other species where elongated etiolated organs often have a reduced diameter due to cellular allocation trade-offs (Le et al. 2005; Zeng et al. 2023). In *Arabidopsis*, rapid hypocotyl elongation is frequently accompanied by cell-wall thinning, particularly in inner tissues, demonstrating that axial expansion can outpace radial reinforcement (Derbyshire et al. 2007). Importantly, despite this trade-off, our field results showed that cultivars with longer hypocotyls improved emergence from depth without reducing early-season biomass, suggesting that elongation rather than early shoot mass is the limiting factor under deep sowing (Harker et al. 2012; Nelson et al. 2022). However, it is possible that thicker stems may be advantageous in some contexts, for example in overcoming physical barriers such as crusted soil surfaces, which were minimised in this study through careful management and hand sowing of the field experiments. Rebetzke et al. (2004) reported coleoptile CSA was associated with larger embryo size in wheat and negatively correlated with coleoptile length, such that increasing seed size at sowing would allow for wider CSA while maintaining greater coleoptile length. In our canola experiments, we restricted the size range of seeds to reduce the influence on seed size on hypocotyl traits. However, we have observed in other experiments that seed size is often positively correlated with CSA and to a lesser extent hypocotyl length. Therefore, sowing of larger canola seed by growers may promote longer hypocotyls together with larger CSA. However, further field experimentation is required to determine the practical relevance of stem thickness for effective seedling emergence, especially in physically restrictive soils (Goward et al. 2026).

We identified significant relationships between hypocotyl traits and seed composition, most notably a positive correlation between hypocotyl length and oil content (Table S2). This could be due to increased energy available for the seedlings to sustain vigorous hypocotyl elongation. Irrespective of the cause, this is a useful trait correlation in that increased oil content is a universal breeding target for canola, which is expected to have a positive influence on hypocotyl length and emergence from deep sowing.

### Genetic control is polygenic with temperature-specific signals

A GWAS identified six significant SNP loci explaining modest effects on hypocotyl length (0.61 to 1.98 cm per locus; Table 4) which is consistent with the well-documented polygenic architecture of early-seedling traits in canola (Basunanda et al. 2010; Luo et al. 2017). Comparison to these two studies indicates only one possible locus shared with this study, which was located on chromosome C4 with a similar effect size (1.65 cm; Basunanda et al. 2010). However, the peaks of these QTL are approximately 14 Mb apart and may be unconnected.

The lack of overlap in significant marker-trait associations between 15 °C and 22 °C treatments despite stable phenotypic ranking may reflect temperature-specific regulatory networks, insufficient statistical power, or QTL × environment interactions, a common challenge in GWAS of complex traits (Lipka et al. 2012). The combination of high heritability and polygenic control suggests that genomic selection may provide opportunity to accumulate favourable alleles at scale in a commercial breeding program. Multi-temperature model training may enable aggregation of small allelic effects and capture environment-specific variance components in breeding pipelines.

The higher frequency of long hypocotyls among accessions originating from winter-type canola regions (e.g., Germany and Great Britain) and their stronger vernalisation requirements parallels findings that traits underlying early-seedling vigour, cold adaptation and vernalisation responsiveness often share regulatory pathways (Auge et al. 2019). Such associations imply either pleiotropy or linkage between developmental regulators and cell/tissue elongation traits. It is highly suggestive that one of the significant SNP markers associated with hypocotyl length was located in the well-known vernalisation gene *VRN1* (Table 4). Further investigation is required to confirm the biological significance of this suggestive association.

The SNP marker on chromosome A03 was located in a *HVA22*-like gene, providing a plausible link between hypocotyl length variation and hormone-regulated germination processes. HVA22-like proteins are induced by abscisic acid (ABA), which generally antagonises gibberellin-mediated germination and early seedling growth. In dormant seeds or under stress, elevated HVA22 activity has been associated with restricted vesicular trafficking and delayed mobilisation of seed storage reserves, whereas favourable conditions and gibberellin signalling promote germination partly through removal of this constraint (Shen et al. 2001; Guo and Ho 2008). Although the direction of effect at this locus requires functional validation, its association with hypocotyl length suggests that allelic variation in ABA–GA balance or downstream trafficking processes may contribute to differences in early seedling elongation capacity.

### Breeding and agronomic implications

Current and historic Australian cultivars generally emerged poorly from deep sowing compared with several long-hypocotyl overseas accessions identified here (Fig. 6). This was supported by previous surveys highlighting establishment limitations in modern Australian cultivars (McMaster and Baxter 2019; Nelson et al. 2022). Because deeper sowing into stored moisture is becoming increasingly common with earlier sowing strategies driven by climate change (Fletcher et al. 2016; Lilley et al. 2019), the ability to reliably emerge from 5 cm depth offers a meaningful agronomic advantage. Historical studies have shown that deep sowing improves stand survival in hot, dry seed-beds but is constrained by insufficient elongation capacity (Harker et al. 2012; Malhi and Gill 2004). Our results confirm that breeding canola with longer hypocotyls could make such practices more reliable.

The phenotyping method used here—dark-grown trays, controlled temperature, sampled once at 230°Cd—achieved high repeatability and produced values predictive of field performance. Together with strong genetic control, the phenotyping in controlled environment and field satisfy key requirements for adoption by breeding programs (Nelson et al. 2022) and mirrors approaches used in wheat long-coleoptile breeding pipelines (Zhao et al. 2022).

### Genotype × environment patterns and implications for deployment

The emergence ratio (5 cm vs 2 cm sowing depths) exhibited significant G×E, with strong site-to-site consistency in some regions and contrasting outcomes in others. Such patterns are expected given that soil properties and surface conditions alter the mechanical barrier to emergence, and small-seeded species are particularly sensitive to crusting and compaction effects (Brill et al. 2016; Harker et al. 2012). Poorer correlations at the nutrient-deficient sand site in this study align with findings that nutrient stress can reduce early biomass and vigour independent of hypocotyl elongation (Nelson et al. 2022). Further, greater soil disruption at 5 cm may disturb soil to increase water loss particularly where soil moisture is marginal. These results emphasise that long-hypocotyl cultivars will deliver their greatest benefit when paired with agronomic practices that ensures uniform soil cover and minimal crusting and moisture loss —similar to the variety × management interplay documented in deep-sown wheat (Zhao et al. 2022). The often-confounding factors of temperature, soil type, compaction and moisture content are difficult to distinguish in conventional field experiments. Goward et al. (2026) recently reported the development of a lab-based methodology that enabled precise control of these interacting factors. We are currently using this methodology to understand how these soil factors influence the expression of the long hypocotyl trait.

Furthermore, the results of our lab and hand sown field experiments need to be further validated in the field using commercial machinery. Hand sowing provides a seed bed with limited disturbance below the seed and light compaction. In field sown machinery there is significant disturbance of soil below the seed and press wheels are used to ensure seed-soil contact. This can lead to markedly different seed beds to those tested here. The use of high press wheel pressure may produce high soil strengths above the seed and limit emergence from depth.

## Conclusions

Together, these results establish long hypocotyl length as a heritable, field-relevant trait to improve canola seedling emergence from deeper sowing. The study provides breeding-ready high-throughput phenotyping methods, identifies candidate loci underlying genetic variation, and demonstrates large benefits in greater field emergence with deep sowing. As climates continue warming and early sowing into dry seed-beds becomes more common (Fletcher et al. 2016; Kirkegaard et al. 2016; Lilley et al. 2019), incorporating long-hypocotyl genetics into modern canola cultivars offers a promising path to more resilient canola establishment. Further work needs to incorporate these traits into modern well adapted cultivars. This is the focus of ongoing research.

## Supplementary Information

Below is the link to the electronic supplementary material.

Supplementary file1 (PPTX 2077 KB)

Supplementary file2 (XLSX 108 KB)

## Data Availability

Full summary data are provided in the supplementary files accompanying this manuscript.
